# Off-Target Effects of P2Y12 Receptor Inhibitors: Focus on Early Myocardial Fibrosis Modulation

**DOI:** 10.3390/ijms242417546

**Published:** 2023-12-16

**Authors:** Francesca Lofrumento, Natasha Irrera, Roberto Licordari, Silvia Perfetti, Enrica Nasso, Paolo Liotta, Giovanni Isgrò, Victoria Garcia-Ruiz, Francesco Squadrito, Scipione Carerj, Gianluca Di Bella, Antonio Micari, Francesco Costa

**Affiliations:** 1 Department of Clinical and Experimental Medicine, Policlinic “G. Martino”, University of Messina, 98122 Messina, Italy; franci.lofru@gmail.com (F.L.); robertolicordari@gmail.com (R.L.); silvia.perfetti@hotmail.it (S.P.); enrica@nasso.it (E.N.); paololiotta94@gmail.com (P.L.); giovanni_isgro@alice.it (G.I.); francesco.squadrito@unime.it (F.S.); scipione.carerj@unime.it (S.C.); gianluca.dibella@unime.it (G.D.B.); 2 Cardiology Department, GIOMI Hospital, 98165 Messina, Italy; mavigaru@gmail.com; 3 BIOMORF Department, Policlinic “G. Martino”, University of Messina, 98122 Messina, Italy; antonio.micari@unime.it (A.M.); dottfrancescocosta@gmail.com (F.C.)

**Keywords:** myocardial infarction, off-target effect, molecular pathways, replacement fibrosis, early modulation

## Abstract

Several studies have demonstrated that, beyond their antithrombotic effects, P2Y12 receptor inhibitors may provide additional off-target effects through different mechanisms. These effects range from the preservation of endothelial barrier function to the modulation of inflammation or stabilization of atherosclerotic plaques, with an impact on different cell types, including endothelial and immune cells. Many P2Y12 inhibitors have been developed, from ticlopidine, the first thienopyridine, to the more potent non-thienopyridine derivatives such as ticagrelor which may promote cardioprotective effects following myocardial infarction (MI) by inhibiting adenosine reuptake through sodium-independent equilibrative nucleoside transporter 1 (ENT1). Adenosine may affect different molecular pathways involved in cardiac fibrosis, such as the Wnt (wingless-type)/beta (β)-catenin signaling. An early pro-fibrotic response of the epicardium and activation of cardiac fibroblasts with the involvement of Wnt1 (wingless-type family member 1)/β-catenin, are critically required for preserving cardiac function after acute ischemic cardiac injury. This review discusses molecular signaling pathways involved in cardiac fibrosis post MI, focusing on the Wnt/β-catenin pathway, and the off-target effect of P2Y12 receptor inhibition. A potential role of ticagrelor was speculated in the early modulation of cardiac fibrosis, thanks to its off-target effect.

## 1. Introduction

Ischemic heart disease holds the foremost position as the primary contributor to mortality from cardiovascular disease (CVD) [1]. Furthermore, it constitutes the predominant underlying cause of heart failure on a global scale [2]. Diverging from other tissues, the myocardium demonstrates a markedly limited ability to regenerate in the aftermath of injuries. Consequently, necrotic cardiomyocytes are replaced by fibrotic scar tissue in the cardiac repair process, which can lead to an adverse cardiac remodeling [3]. Different cell types, including fibroblasts and macrophages, are involved in this process and play a pivotal role by releasing a wide array of mediators (i.e., cytokines) that regulate the activation of multiple molecular pathways, such as the Wnt/β-catenin pathway, involved in cardiac fibrosis. For this reason, the modulation of these pathways might be effective in promoting the replacement of fibrosis in reactive tissue [3,4]. Dual antiplatelet therapy (DAPT), consisting of the combination of a platelet P2Y12 receptor inhibitor and aspirin, is the cornerstone of treatment for patients with acute coronary syndromes (ACS) requiring percutaneous coronary interventions (PCI) [5]. P2Y12 receptor activation, a platelet purinergic receptor for adenosine 5′-diphosphate (ADP), significantly contributes to the arterial thrombosis process [6]. Therefore, different P2Y12 inhibitors have been developed, from ticlopidine, the first thienopyridine, to the more potent non-thienopyridine derivatives, such as ticagrelor, in order to provide a beneficial antithrombotic effect [7]. Moreover, in recent years, non-platelet-related off-target P2Y12 receptor inhibitor effects have also been explored—from the impact of P2Y12 receptor inhibition on other cell types, including endothelial cells (EC) and immune cells [8,9,10], to the adenosine-mediated effects of ticagrelor [11]—unraveling a possible impact of these drugs on molecular pathways related to heart failure. The aim of this review was to explore the off-target effects of P2Y12 receptor inhibitors and their potential role on myocardial fibrosis and on the related pathways, such as the Wnt/β-catenin signaling pathway.

## 2. Off-Target Effects of P2Y12 Receptor Inhibitors

The off-target effects of P2Y12 receptor inhibitors have been recently investigated, thus expanding their clinical benefits beyond mere platelet inhibition and arterial thrombosis prevention.

### 2.1. P2Y12 Platelet Receptors and Inhibitors

Among extracellular nucleotides, ADP assumes a central role in platelet function and the initiation of thrombus formation. Platelets express two purinergic receptors for ADP, namely P2Y1 and P2Y12, which are coupled to Gq (G-protein q) and Gi (G-protein i), respectively [7,8,9]. Of these, P2Y12 holds particular significance. Stimulation of P2Y1 by ADP leads to the activation of the glycoprotein (GP) IIb/IIIa receptor, resulting in calcium mobilization, alterations in platelet shape, and transient platelet aggregation. In contrast, activation of P2Y12 triggers the GP IIb/IIIa receptor, culminating in platelet degranulation, thromboxane production, and prolonged platelet aggregation. This, in turn, establishes a positive feedback loop that contributes significantly to the formation of arterial thrombi [12,13]. This thrombotic process represents a hallmark of myocardial infarction (MI) pathogenesis, underscoring the identification of P2Y12 as a therapeutic target for the management and prevention of arterial thrombosis [7].

Ticlopidine was the first P2Y12 antagonist approved by the Food and Drug Administration (FDA) in 1991 [7]. Subsequently, several P2Y12 inhibitors have been introduced into clinical practice. These P2Y12 inhibitors are categorized into two classes of drug: the thienopyridines, comprising clopidogrel, prasugrel, and ticlopidine, and the non-thienopyridine derivatives such as ticagrelor, cangrelor, and elinogrel. Thienopyridines are administered orally as prodrugs and undergo hepatic and intestinal metabolism to yield active metabolites, which subsequently bind covalently to the P2Y12 receptor, thereby inducing irreversible platelet inhibition throughout their lifespan. The production of these active metabolites, particularly in the case of clopidogrel, relies on cytochrome P450 (CYP450) enzymes in the liver. However, clopidogrel is accompanied by several limitations, including a delayed onset of action, variability in response, resistance to the drug, moderate inhibition of platelet aggregation (ranging from 33% to 64%), and potential drug interactions due to the involvement of CYP450 enzymes and genetic variations [11,12]. In response to these challenges, the third-generation thienopyridine, prasugrel, was developed. In contrast to ticlopidine and clopidogrel, prasugrel initially undergoes metabolism by an intestinal esterase. This initial step is followed by a single-step activation process dependent on CYP450 enzymes. This distinctive metabolic pathway results in a swifter onset of action, more potent inhibition of platelet aggregation (ranging from 50% to 80%), reduced resistance to the drug, and a lower likelihood of drug interactions. However, it is worth noting that prasugrel is associated with an increased risk of bleeding [11,12]. The second category, consisting of non-thienopyridine derivatives, does not necessitate hepatic bioactivation and delivers reversible P2Y12 receptor inhibition [6]. Among these, ticagrelor stands out as an oral direct-acting P2Y12 inhibitor. It boasts a rapid onset of action within two hours and exerts a substantial inhibitory effect on platelet aggregation, ranging from 60% to 90%. Additionally, the active metabolite, AR–C124910XX, is generated through hepatic metabolism of ticagrelor and contributes to one-third of its antiplatelet effects. Based on the findings of the phase III Platelet Inhibition and Patient Outcomes (PLATO) trial and the Trial to Assess Improvement in Therapeutic Outcomes by Optimizing Platelet Inhibition with Prasugrel Thrombolysis In Myocardial Infarction 38 (TRITON-TIMI 38), it is recommended, as the primary strategy, to employ dual antiplatelet therapy (DAPT) comprising both aspirin and a potent P2Y12 receptor inhibitor, such as prasugrel or ticagrelor, for patients with acute coronary syndrome (ACS) [5,14,15]. The use of clopidogrel, which offers less effective and more variable platelet inhibition, should be limited to situations where prasugrel or ticagrelor are contraindicated or unavailable, or in specific instances where there is a high risk of bleeding (according to criteria such as ≥1 major or ≥2 minor ARC-HBR criteria) [10,16]. Additionally, the consideration of clopidogrel may be appropriate for elderly patients (i.e., those aged ≥ 70 years) [5,10,17].

### 2.2. Non-Platelet P2Y12 Receptors

In addition to their established hemostatic functions, platelets are increasingly recognized for their substantial involvement in modulating inflammatory and immune responses. This recognition is attributable to their interaction with immune cells through the exposure of membrane proteins such as P-selectin and CD40 ligand (cluster of differentiation 40), as well as their release of inflammatory mediators, including cytokines and chemokines [8,18,19].

**Figure 1:**
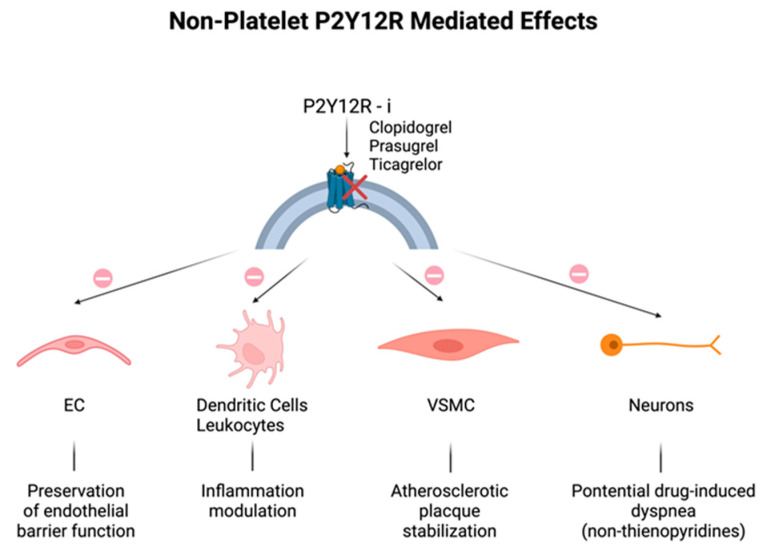
Non-platelet P2Y12 receptor (P2Y12R)-mediated off-target effects: additional effects of all P2Y12R inhibitors on different cell types. (**Top**): P2Y12 receptor blockade (red cross) by clopidogrel, prasugrel, and ticagrelor. (**Bottom**): effects (arrows) of PY12R inhibition (prohibition symbol) on endothelial cells (ECs), immune cells, vascular smooth muscle cells (VSMCs), and neurons.

Historically, P2Y12 receptors have been recognized to be specific in platelets, but the discovery of their expression in other cells, including endothelial cells (ECs), vascular smooth muscle cells (VSMCs), immune cells, dendritic cells, and neurons could explain the additional effects related to their modulation (Figure 1) [8,20,21]. As for ECs, disruption of endothelial barrier function was attenuated by the increase in EC cyclic adenosine monophosphate (cAMP) levels, because of the P2Y12 receptor blockade [22,23]. P2Y12 receptor activation on VSMCs may induce an inflammatory state closely associated with atherosclerotic plaque instability [24], that may be mitigated by P2Y12 receptor inhibitors. Lastly, neuronal P2Y12 receptors might seem to be the mediators of drug-induced dyspnea observed in patients treated with non-thienopyridine derivatives [25]; in fact, repeated dosing of reversible P2Y12 receptor inhibitors, like ticagrelor, ensures a continuous blockage of neural P2Y12 receptors, in contrast to temporary and transient clopidogrel interaction, due to the fact that, unlike platelets, nucleated cells exhibit the capacity to promptly substitute the suppressed receptors [11].

**Figure 2:**
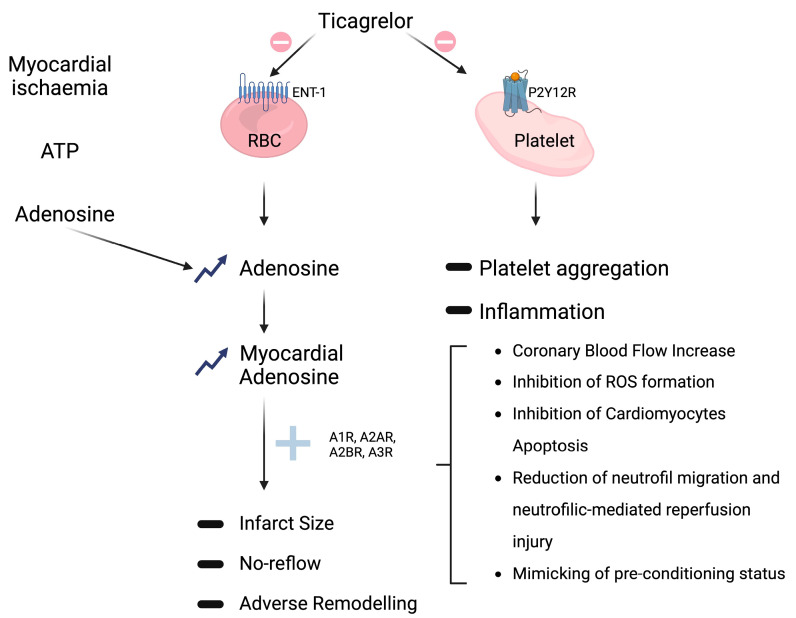
Non-P2Y12-mediated off-target effects of ticagrelor. (**Top right**): conventional platelet PY12R inhibition (prohibition symbol) resulting (arrows) in reduction (minus sign) of platelet aggregation and inflammation (see Section 2.2). (**Middle**): ticagrelor off-target inhibition (prohibition symbol) of adenosine reuptake via sodium-independent equilibrative nucleoside transporters 1 (ENT1) on red blood cells (RBCs) that leads (arrows) to both systemic and tissue adenosine levels elevation (upward arrows); adenosine interaction (plus sign) with its receptors (A1R, A2AR, A2BR, and A3R) culminates, through different possible mechanisms (square bracket), in multiple favorable outcomes, like reduction (minus sign) of infarct size, no-reflow, and adverse remodeling. (**Top left**): adenosine production (arrows) from adenosine triphosphate (ATP) degradation by CD39 and CD73 enzymes in response to hypoxia and tissue damage.

Therefore, the impact and clinical advantages of P2Y12 receptor antagonism may extend beyond merely platelet inhibition and arterial thrombosis prevention. Additional potential consequences encompass the attenuation of the pro-inflammatory effects of activated platelets (Figure 2) and the impact of P2Y12 receptor inhibition on various other cell populations [8,20,23].

### 2.3. Non-P2Y12-Mediated Effects

In the PLATO (Platelet Inhibition and Patient Outcomes) trial, ticagrelor demonstrated superiority over clopidogrel in prevention of cardiovascular death, myocardial infarction, or stroke, with incidence rates of 9.8% compared to 11.7%, resulting in a relative risk reduction of 16% [18]. An additional positive effect on all-cause mortality, which was reduced by 24% by ticagrelor compared to clopidogrel, has been observed. Considering that such mortality benefit was not observed in the pivotal trial of prasugrel, a potent P2Y12 inhibitor with a comparable P2Y12 inhibitory effect, such mortality benefit has been speculated to be linked to the additional non-P2Y12-mediated off-target mechanisms of the drug [19].

The most extensively documented off-target mechanism pertains to ticagrelor’s inhibition of adenosine reuptake by red blood cells, accomplished via sodium-independent equilibrative nucleoside transporter 1 (ENT1). This action leads to the elevation of both systemic and tissue adenosine levels (Figure 2) [6,20,23]. Van Giezen et al. illustrated that ticagrelor inhibits adenosine reuptake in erythrocytes, resulting in an elevation of adenosine levels in the bloodstream, accompanied by a notable, dose-dependent enhancement of adenosine-mediated coronary blood flow [26]. Furthermore, ticagrelor was also able to reduce the size of the infarction area, an effect not observed following clopidogrel treatment [27]. Vilahur et al. provide additional evidence supporting the non-P2Y12-mediated hypothesis—ticagrelor significantly reduced the infarct size compared to clopidogrel—an effect substantiated through the evaluation of troponin-I levels and histopathological analysis. Moreover, in comparison to clopidogrel, ticagrelor led to a significant reduction in myocardial edema and activated both AMP-activated protein kinase signaling and cyclooxygenase-2. Importantly, the advantageous outcomes associated with ticagrelor in comparison to clopidogrel were hindered by the A1/A2 receptor antagonist 8-p-sulfophenyl theophylline. Therefore, these studies underline that ticagrelor may play an important cardioprotective role by decreasing necrotic injury and edema via adenosine-dependent mechanisms in addition to its antiplatelet role [28,29]. Regarding human investigations, it was observed that the maintenance dose of ticagrelor led to an augmentation of adenosine-induced coronary blood flow velocity in non-ST-elevation ACS patients undergoing percutaneous coronary intervention (PCI) [30]. Furthermore, Bonello et al. provided evidence of ticagrelor significantly elevating adenosine plasma levels in comparison with clopidogrel in ACS patients. In the same study, it was noted that serum from patients treated with ticagrelor, but not clopidogrel, exhibited the capacity to inhibit the uptake of exogenous adenosine by erythrocytes in vitro [31]. A double-blind, placebo-controlled, randomized study involving 40 healthy male subjects also showed that a single 180 mg loading dose of ticagrelor increased adenosine-induced coronary blood flow velocity. These beneficial effects, as well as the higher rate of dyspnea and bradycardia observed following ticagrelor administration, were probably related to higher adenosine levels, which were reversed by theophylline, an adenosine receptor antagonist, thus suggesting that all these effects are mediated by adenosine receptors [32].

These findings affirm that oral administration of ticagrelor may be sufficient to impede the cellular uptake of adenosine, thus extending its half-life and elevating its concentration, thereby enhancing its cardioprotective effects. This off-target effect associated with ticagrelor has the potential to ameliorate endothelial function, although it should be noted that the current body of evidence remains somewhat limited [6,33]. In light of these considerations, the HI-TECH (Hunting for the Off-target Properties of Ticagrelor on Endothelial Function and Other Circulating Biomarkers in Humans) trial was undertaken to investigate the off-target effects of ticagrelor on endothelial function. This trial marked the third randomized study with this specific objective but was the first to juxtapose ticagrelor with both prasugrel and clopidogrel in a chronic setting, focusing on stabilized post-ACS patients. This approach was adopted to mitigate the potential confounding influences of the natural course of the disease, including the acute inflammatory phase of ACS and tissue ischemia, on the comparison across P2Y12 inhibitors and systemic adenosine plasma levels. In the context of this trial, assessments of endothelial-dependent dilatation, as gauged by the reactive hyperemia index (RHI), and, in a subgroup of patients, flow-mediated dilatation (FMD) of the brachial artery, showed no significant disparities between ticagrelor and prasugrel or clopidogrel at the presently approved regimens. Likewise, systemic adenosine plasma levels and vascular biomarkers exhibited no noteworthy distinctions at any of the time points measured [34]. Conversely, a post hoc analysis of the PLATO trial unveiled a notable association between ticagrelor, in comparison with clopidogrel, and reduced morbidity and mortality attributable to pulmonary infection and sepsis [35]. These observations stem from the combined effect of adenosine, which plays an augmenting role in modulating the inflammatory response, and the anti-inflammatory impact of P2Y12 receptor inhibition, as explained earlier. The potential of ticagrelor to confer anti-inflammatory effects through adenosine was also demonstrated in vitro, where ticagrelor was found to enhance adenosine-induced neutrophil migration [36]. However, this beneficial effect did not manifest in the PEGASUS trial, where there was a numerical increase in deaths attributed to infections in the 90 mg ticagrelor group when compared to the 60 mg ticagrelor and placebo groups [37]. It is important to note that these divergent outcomes between the PLATO and PEGASUS trials may be attributed to the distinct patient populations under study. For instance, the anti-inflammatory effect might have held particular significance for patients who had undergone coronary artery bypass grafts (CABGs), as they might have been more vulnerable to infections. In the PLATO trial, ticagrelor demonstrated a 50% reduction in mortality compared to clopidogrel in CABG patients [35]. In the PEGASUS trial, which included a more stable patient cohort, fewer individuals would have undergone CABG, potentially explaining the differences in infection or sepsis-related mortality. More recently, the Targeting Platelet–Leukocyte Aggregates in Pneumonia with Ticagrelor (XANTHIPPE) study reported an association between ticagrelor and improved lung function in patients hospitalized for pneumonia [38]. Notably, ticagrelor’s antimicrobial activity was recently established in mouse models, although it differs from the pharmacokinetics observed in humans [39]. Therefore, further investigations are needed. In conclusion, a debate about the potential off-target adenosine-mediated effects of ticagrelor is still ongoing.

Importantly, it is noteworthy that only ticagrelor, and not its metabolite AR-C124910XX, exhibits a substantial capacity for inhibiting ENT1 [40]. Additionally, aside from its ENT1 inhibitory effects, ticagrelor has demonstrated the induction of ATP release from human erythrocytes in vitro; however, the translation of this phenomenon to in vivo settings remains uncertain. Both of these mechanisms would act synergistically to further elevate adenosine concentration—ATP undergoes rapid conversion into AMP, facilitated by ectonucleoside triphosphate diphosphohydrolase 1 (CD39), ultimately leading to adenosine production via the action of ecto-5′-nucleotidase (CD73), as previously emphasized by Cattaneo et al. [11].

#### Adenosine-Mediated Effects

Adenosine is locally generated in response to hypoxia and tissue damage, a process involving the degradation of adenosine triphosphate (ATP) and adenosine diphosphate (ADP) by CD39 and CD73 enzymes. Subsequently, adenosine is swiftly internalized through ENT1. The inhibition of ENT1 by ticagrelor leads to an intensified response to adenosine, which acts through interactions with adenosine receptors belonging to the G-protein-coupled receptor family, including (i) A1R and A3R, coupled to the inhibitory G protein (Gi), which inhibits adenylyl cyclase, thereby reducing intracellular cAMP, and (ii) A2AR and A2BR, coupled to the stimulatory G protein (Gs), which activates adenylyl cyclase, resulting in increased intracellular cAMP levels [11,14]. Pre-clinical investigations have reported multiple favorable outcomes associated with the use of adenosine agonists, positioning adenosine as a potential cardioprotective agent during acute myocardial infarction (AMI) (as depicted in Figure 2) [9,41,42]. Specifically, an increase in myocardial adenosine levels has been linked to augmented coronary blood flow, reduced expression of inflammatory markers, diminished fibrosis, reduced infarct size, decreased edema, and enhanced tissue remodeling [6,9,43]. Furthermore, adenosine has demonstrated the ability to attenuate and potentiate ischemic preconditioning, thereby conferring protection against subsequent reperfusion injury [9,44].

Da Silva and colleagues have made a pioneering contribution by showcasing the effectiveness of orally administered LASSBio-294, an A2A-adenosine receptor agonist, in preventing the advancement of left ventricular (LV) dysfunction following myocardial infarction (MI) within a preclinical model characterized by preexisting hypertension [45]. Notably, the same authors had previously established the efficacy of this compound in preventing heart failure in normotensive animals when administered intraperitoneally [46]. LASSBio-294 effectively mitigated fibrotic and inflammatory responses, by reducing LV collagen deposition and tumor necrosis factor α expression [45]. This discovery points to the potential role of cAMP-dependent signaling, regulated via A2AR activation, in modulating cardiac remodeling and the progression of heart failure by mitigating fibroblast proliferation and collagen synthesis [45,47]. Additionally, a study conducted in China in 2022 shed light on the beneficial effects of Shenfu injection (SFI), a commonly employed treatment for cardiac dysfunction in China. The study illustrated that SFI attenuated cardiac fibrosis through the activation of adenosine A2A receptors (A2ARs) in a rat model of myocardial ischemia-reperfusion (MI/R) [48].

Therefore, the clinical use of A2A receptor agonists could be a potential alternative treatment for heart failure, in addition to the conventional anti-remodeling therapies. In fact, considering the modest efficacy of traditional therapies in terms of cardiovascular morbidity and mortality [49], it is worth exploring new pharmacological targets for heart-failure treatment.

## 3. Myocardial Fibrosis

Myocardial fibrosis is the common denominator of different cardiac conditions, from hypertension to myocardial infarction to cardiomyopathies. Focusing on post-MI fibrosis, prolonged ischemic injury with irreversible cellular damage cause cardiomyocyte (CM) necrosis resulting in fibrotic scar formation, which replaces the lost cells [11]. This is principally caused by the post-mitotic nature of CM that limits the regenerative myocardium potential in adult mammalian tissue [50].

The initial stages of the wound healing process entail the activation and recruitment of immune cells in response to signals of damage within the necrotic region. This region, characterized by the presence of dead cells and interstitial debris, undergoes clearance through processes such as phagocytosis and proteolysis, marking the *inflammatory phase*. Subsequently, neutrophils can stimulate the anti-inflammatory and pro-fibrotic activities of macrophages, thus counteracting inflammation and promoting the reparative process. This *reparative phase* is defined by the production of the extracellular matrix (ECM) [51]. The subsequent stages involve cell proliferation followed by a maturation phase of the scar, during which ECM structures undergo crosslinking, contributing to the stabilization of the scar tissue. This phase is accompanied by the deactivation and eventual death of reparative cells, as well as the removal of matricellular proteins. The equilibrium between the inflammatory and reparative phases plays a pivotal role in regulating the healing process and significantly impacts the quality of the resulting scar. Disproportionate collagen deposition can lead to excessive scar stiffness, thereby affecting cardiac compliance and both diastolic and systolic function. Conversely, an abnormal inflammatory phase can result in inadequate collagen deposition and cross-linking, rendering the scar susceptible to dilation, adverse cardiac remodeling, and the risk of wall rupture [6,52]. Furthermore, fibrotic scarring and interstitial fibrosis can exert influences on myocardial electrical activity, potentially predisposing individuals to life-threatening ventricular arrhythmias [53].

### 3.1. Molecular Signaling Pathways

Numerous molecular signaling pathways play integral roles in the process of cardiac fibrosis, with one of the most prominent pathways being the transforming growth factor-beta (TGF-β) pathway. TGF-β is widely recognized as the primary driver of fibroblast proliferation and their differentiation into myofibroblasts, and it serves as the principal inducer of collagen production [52]. The binding of TGF-β to the TGF-β type II receptor forms a stable and active complex that subsequently phosphorylates the TGF-β type I receptor. Activation of downstream cascades is then initiated by the kinase activity of the TGF-β type I receptor, primarily involving members of the Smad family of proteins [54]. It is important to note that the TGF-β signaling cascade can also be activated independently of the Smad pathway [55]. However, it should be acknowledged that multiple signaling pathways contribute to the complex orchestration of cardiac fibrosis.

#### Wnt/β-Catenin Pathway

**Figure 3:**
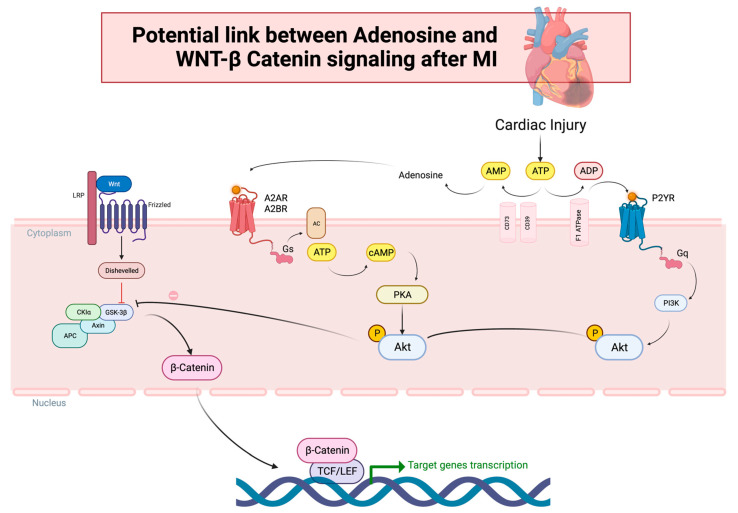
Potential link between adenosine and Wnt/β-catenin signaling after MI. (**Left**): the canonical Wnt/β-catenin pathway. When Wnt glycoproteins bind to the specific frizzled (Fz) receptor and the co-receptor lipoprotein receptor-related protein 5/6 (LRP5/6), the “destruction complex” composed of axin, adenomatosis polyposis coli (APC), glycogen synthase kinase 3 (GSK3), and casein kinase 1 alfa (CK1a) is recruited to the cell membrane where it is inhibited (red line) by the protein Dishevelled, so that cytosolic β-catenin can translocate (arrows) into the nucleus and regulate the expression of specific genes interacting with transcription factors TCF/LEF (T cell factor/lymphocyte enhancer factor-1). (**Middle**): Adenosine, a product of adenosine triphosphate (ATP) degradation by CD39 and CD73 enzymes (arrows) in response to cardiac injury, can stimulate (arrow) its receptor A2AR and A2BR, coupled to the stimulatory G protein (Gs), which activates adenylyl cyclase (AC), resulting (arrow) in increased intracellular cyclic adenosine monophosphate (cAMP) levels, subsequently activating (arrow) protein kinase A (PKA). Then, PKA phosphorylates (P symbol) and activates (arrow) protein kinase B (Akt), which further phosphorylates (arrow) GSK3, inhibiting (prohibition symbol) the “destruction complex”, thus facilitating β-catenin translocation (arrows) into the nucleus. Right: Akt activation can also be induced (arrows) by the phosphatidylinositol-3-kinase (PI3K) pathway, which can, itself, be triggered by P2YR receptor activation by adenosine diphosphate (ADP), resulting from ATP catalysis by F1 ATPase enzyme.

The Wnt/β-catenin pathway constitutes a protein family crucial in embryonic development. In the adult heart, it has been traditionally regarded as quiescent [56,57]. However, in recent years, it has been shown that it is reactivated in response to myocardial injury, assuming the role of a pro-fibrotic signal [52,57]. The Wnt signal engages various cascades, encompassing (a) the canonical Wnt/β-catenin pathway, (b) the non-canonical Wnt/planar cell polarity (PCP) pathway, and (c) the WNT/Ca^2+^ pathway. Multiple Wnt glycoproteins can elicit activation of both the canonical and non-canonical pathways, thus fostering the fibrosis process in either case [53]. Of particular note, the canonical Wnt pathway operates through β-catenin-dependent signaling. Without Wnt signaling, β-catenin is confined within the cytoplasmic compartment, under the governance of a “destruction complex” composed of axin, adenomatosis polyposis coli (APC), protein phosphatase 2A (PP2A), glycogen synthase kinase 3 (GSK3), and casein kinase 1 (CK1). This complex orchestrates β-catenin degradation through a series of phosphorylation events. Conversely, when Wnt glycoproteins bind to the specific frizzled (Fz) receptor and the co-receptor lipoprotein receptor-related protein 5/6 (LRP5/6), cytosolic β-catenin undergoes stabilization and translocation into the nucleus, where it interfaces with transcription factors to regulate the expression of specific genes [9,52,57,58]. Apart from Wnt ligands, various other molecules, including protein kinase B (Akt), can stimulate β-catenin translocation. The activation of Akt can be initiated by adenosine, with adenosine A2AR stimulation elevating cAMP levels, subsequently activating protein kinase A (PKA), which leads to Akt phosphorylation and activation. Akt further phosphorylates GSK3, a component of the β-catenin “destruction complex”, thus facilitating β-catenin translocation into the nucleus. Moreover, Akt activation can also be induced by the phosphatidylinositol-3-kinase (PI3K) pathway, which can itself be triggered by A2AR, A2BR, and P2YR receptor activation (Figure 3). In particular, PI3K recruits phospholipid-dependent kinase 1 (PDK1), via phospholipid phosphorylation, ultimately culminating in Akt activation [52,59,60].

Recent investigations have unveiled a significant interplay between GSK-3β, the β-catenin pathway, and the canonical TGF-β1-SMAD-2/3 cascade, which collectively govern the intensity and the length of profibrotic signaling [52,61]. Emerging evidence points to the anti-fibrotic role played by GSK-3β through its direct interaction with SMAD-3, where it negatively modulates SMAD-3 protein stability and enzymatic activity. Guo et al. were the pioneering researchers who elucidated the regulatory mechanism by which GSK-3β modulates the protein stability of SMAD-3, contributing to the precise modulation of TGF-β1 signaling [62]. This discovery has been further corroborated by in vivo studies demonstrating the direct interaction between GSK-3β and SMAD-3 in cardiac fibroblasts of mouse and human hearts. Deletion of GSK-3β in cardiac fibroblasts led to SMAD-3 hyperactivation, resulting in an excessive fibrotic response and adverse ventricular remodeling [63]. In addition, GSK-3β constitutes an integral component of the Wnt/frizzled signal transduction pathways, governing the balance between the cytoplasmic deposition and nuclear translocation of β-catenin. GSK-3β deletion can promote β-catenin nuclear translocation, thereby activating the β-catenin-dependent gene program [61]. Furthermore, Blyszczuk et al. have illustrated the crucial significance of the interplay between β-catenin and TGF-β1 signaling in the advancement of myocardial fibrosis and the differentiation of cardiac fibroblasts into myofibroblasts [64]. The prevention of TGF-β1-induced differentiation of cardiac fibroblasts into myofibroblasts was effectively achieved through the inhibition of Wnt signaling by secreted frizzled-related protein 2 (sFRP2) or Wnt-C59. Moreover, the administration of TGF-β1 was adequate to induce Wnt secretion and activate the Wnt pathway in isolated cardiac fibroblasts (CFs). These findings underscore the necessity for co-activation of TGF-β1 and Wnt signaling pathways in the differentiation of CFs into the myofibroblast phenotype in diseased hearts [64]. Consequently, the signaling network involving GSK-3β, β-catenin, and TGF-β1/SMAD-3 has arisen as a potent regulator of myocardial fibrosis.

Nevertheless, conflicting evidence persists concerning the role of Wnt signaling in cardiac repair. While most studies underscore the beneficial impact of inhibiting Wnt signaling pathways in reducing cardiac fibrosis, using Wnt antagonists such as secreted frizzled-related protein (Sfrp2) [65,66], there is a nuanced view within the literature, revealing a biphasic effect of the Wnt/β-catenin pathway. When focusing on the early post-myocardial infarction (MI) phase, quantitative analyses of Wnt protein expression have unveiled notable upregulation of Wnt-2, Wnt-4, Wnt-10b, and Wnt-11 five days after MI [67]. Wnt-1 exhibited upregulation from days 1 to 14 post-MI, whereas Wnt-4 upregulation was observed from days 7 to 14 after MI [68]. Duan et al.’s study [68] shed light on the dynamic response of Wnt1/β-catenin to injury, which serves as a catalyst for the activation of the epicardium and cardiac fibroblasts, crucial for cardiac repair. In fact, Wnt1 is prominently upregulated in the heart following cardiac injury, with initial expression in the epicardium and subsequent expression by cardiac fibroblasts at the site of cardiac injury. The epicardium becomes responsive to Wnt, leading to widespread activation and expansion, thereby instigating epithelial–mesenchymal transition (EMT) in order to activate fibroblasts. These activated fibroblasts are then localized in the subepicardial space, with concurrent upregulation of pro-fibrotic gene expression. Notably, a disruption of downstream Wnt/β-catenin signaling in epicardial cells hampers epicardial expansion and EMT, resulting in a severe compromise of cardiac function after cardiac injury. Furthermore, the impairment of downstream Wnt signaling in cardiac fibroblasts is associated with a prompt deterioration in cardiac function, compromised wound healing, and an early enlargement of the cardiac chambers. Hence, these findings underscore the critical requirement of a pro-fibrotic Wnt1/β-catenin injury response, orchestrated through the activation of the epicardium and cardiac fibroblasts, in preserving cardiac function following acute ischemic cardiac injury [67].

In conclusion, these studies underline that the biological effect of the Wnt/β-catenin pathway is strongly related to the timing and duration of its activation—its early activation is fundamental, contributing to the reparative phase of the cardiac fibrosis post MI. Of course, the modulation of this pathway is necessary to avoid an excessive pro-fibrotic stimulation in the late phase of cardiac fibrosis, which could be deleterious.

## 4. Potential Role of P2Y12 Receptor Inhibitors on Myocardial Fibrosis Modulation

Considering that only ticagrelor has a potential off-target effect mediated by adenosine levels through ENT1 inhibition, it could be speculated that an additional potential positive role of ticagrelor in preventing cardiac remodeling, could be mediated by the adenosine-related effects, also on the Wnt/β-catenin pathway (Figure 2).

HEALING-AMI represents the inaugural human study demonstrating the influence of P2Y12 inhibition on left ventricular (LV) remodeling in patients afflicted with ST-segment elevation myocardial infarction (STEMI). Notably, ticagrelor exhibited superior effects in comparison with clopidogrel concerning LV remodeling. These effects were evident in the reduction in NT-proBNP levels and the amelioration of 3-dimensional echocardiographic parameters, and, impressively, they endured even one month following the occurrence of STEMI. The clinical advantages associated with ticagrelor in STEMI patients may be intricately linked to the facilitation of a more robust recovery from post-myocardial infarction (MI) LV dysfunction, due to adenosine level increase mediated by ticagrelor [69]. However, there are still no studies that have thoroughly evaluated β-catenin expression in ticagrelor-treated patients. The in-depth study of these mechanisms could provide further insights into the potential off-target effects of antiplatelet agents beyond their antithrombotic effect. This might also inform patient-specific strategies to better select specific agents, also based on the heart-failure risk of the single patient.

## 5. Conclusions

Several studies have consistently demonstrated that, beyond their antithrombotic effects, P2Y12 inhibitors may provide additional off-target adenosine-mediated effects. Adenosine may interact and stimulate different pathways involved in cardiac fibrosis, such as the Wnt/β-catenin signaling pathway. The early activation of this pathway is critically required for cardiac function preservation after acute ischemic cardiac injury. Future studies will shed light on this possible off-target effect carried out by P2Y12 inhibitors that could influence the molecular pathways mainly involved in cardiac fibrosis.

## Data Availability

No new data were created or analyzed in this study. Data sharing is not applicable to this article.
